# A Novel *Legionella* Genomic Island Encodes a Copper-Responsive Regulatory System and a Single Icm/Dot Effector Protein Transcriptionally Activated by Copper

**DOI:** 10.1128/mBio.03232-19

**Published:** 2020-01-28

**Authors:** Marika Linsky, Yevgeniya Vitkin, Gil Segal

**Affiliations:** a Department of Molecular Microbiology and Biotechnology, School of Molecular Cell Biology and Biotechnology, George S. Wise Faculty of Life Sciences, Tel-Aviv University, Tel-Aviv, Israel; Pasteur Institute

**Keywords:** *Legionella*, effectors, copper, signal, horizontal gene transfer, two-component system

## Abstract

The intracellular pathogen Legionella pneumophila utilizes the Icm/Dot type IV secretion system to translocate >300 effector proteins into host cells during infection. The regulation of some of these effector-encoding genes was previously shown to be coordinated by several global regulators, including three two-component systems (TCSs) found in all the *Legionella* species examined. Here, we describe the first *Legionella* genomic island encoding a single Icm/Dot effector and a dedicated TCS, which regulates its expression. This genomic island, which we named Lci, undergoes horizontal gene transfer in the *Legionella* genus, and the TCS encoded from this island (LciRS) is homologous to TCSs that control the expression of various metal resistance systems found in other bacteria. We found that the L. pneumophila sensor histidine kinase LciS is specifically activated by copper via a unique, small periplasmic sensing domain. Upon activation by LciS, the response regulator LciR directly binds to a conserved regulatory element and activates the expression of the adjacently located *lciE* effector-encoding gene. Thus, LciR represents the first local regulator of effectors identified in L. pneumophila. Moreover, we found that the expression of the *lciRS* operon is repressed by the Fis1 and Fis3 regulators, leading to Fis-mediated effects on copper induction of LciE and silencing of the expression of this genomic island in the absence of copper. This island represents a novel type of effector regulation in *Legionella*, shedding new light on the ways by which the *Legionella* pathogenesis system evolves its effector repertoire and expands its activating signals.

## Abstract (precis)

Legionella pneumophila is an intracellular human pathogen that utilizes amoebae as its environmental host. The adaptation of L. pneumophila to the intracellular environment requires coordination of expression of its multicomponent pathogenesis system, which is composed of a secretion system and effector proteins. However, the regulatory factors controlling the expression of this pathogenesis system are only partially uncovered. Here, we discovered a novel regulatory system that is activated by copper and controls the expression of a single effector protein. The genes encoding both the regulatory system and the effector protein are located on a genomic island that undergoes horizontal gene transfer within the *Legionella* genus. This regulator-effector genomic island represents the first reported case of local regulation of effectors in *Legionella*. The discovery of this regulatory mechanism is an important step forward in the understanding of how the regulatory network of effectors functions and evolves in the *Legionella* genus.

## INTRODUCTION

Legionella pneumophila is an intracellular human pathogen that multiplies within alveolar macrophages and causes a severe pneumonia known as Legionnaires’ disease (1–3). In the environment, L. pneumophila thrives in many different protozoan cells (4–6), which serve as its training grounds for pathogenesis (7). Inside its eukaryotic host cells, the bacterium remodels its phagosome to generate the *Legionella*-containing vacuole (LCV) (8, 9). Establishment of the LCV depends on the Icm/Dot type IV secretion system, which delivers more than 300 effector proteins, which modulate numerous host-cell functions during infection (10–14). The enormous number of effectors that participate in LCV establishment and the various host cell pathways manipulated by L. pneumophila effectors (15–17) imply that a successful infection will require different levels of coordination among the effectors, including on the level of gene expression.

To date, five regulatory systems have been shown to directly regulate the expression of effector-encoding genes (EEGs) in L. pneumophila: (i) the PmrAB two-component system (TCS) activates the expression of about 40 EEGs (18, 19); (ii) the CpxRA TCS activates or represses the expression of about 30 EEGs and also regulates the expression of several *icm/dot* genes (20–23); (iii) the LetAS-RsmYZ-CsrA regulatory cascade represses the expression of about 40 EEGs during exponential phase (24–31); (iv) two Fis regulators repress the expression of about 20 EEGs during exponential phase (32); and (v) the Fur regulator controls the expression of a single EEG (*mavN*) as well as several other proteins involved in iron acquisition (33–37). In addition, these regulatory systems have been shown to assemble into an interconnected regulatory network using accessory components, such as modulators (AckA-Pta and PTS^Ntr^ [22, 38]) and connectors (LetE and LerC [22, 39, 40]). All the direct regulators of EEGs described above (PmrA, CpxR, CsrA, Fis1, Fis3, and Fur) were found to be present in all the *Legionella* species examined (41, 42), and they function as global regulators that regulate the expression of a large number of genes, including EEGs, scattered throughout the L. pneumophila genome (18–20, 22). Besides global regulators, local regulators that regulate the expression of a small number of adjacent genes are also common in many bacterial systems (43, 44), including pathogenesis-related genes in Vibrio cholerae and Salmonella enterica (45). Local regulators were shown to function as either activators or repressors, and in many cases, they are found in genomic islands together with the genes they regulate (46–49).

Genomic islands are genetic elements acquired via horizontal gene transfer (HGT) that include sets of genes that encode proteins that may be beneficial for the bacteria under certain conditions (50, 51). Many pathogenicity islands have been described in bacteria, some of which contain a large number of genes and encode complete pathogenesis systems (50, 52). For example, the Salmonella enterica pathogenicity island 2 (SPI-2) encodes the complex components of a type III system, effector proteins, and the SsrAB TCS, which functions as a local regulator and coordinates their expression (53). Smaller pathogenicity islands that encode a few proteins are sometimes referred to as islets (50). For example, the Streptococcus pneumoniae RlrA pathogenicity islet encodes the RlrA local transcriptional regulator, which controls the expression of genes located on the same islet that are essential for lung infection (54). Another example is the Listeria monocytogenes virulence gene cluster that encodes phospholipases, listeriolysin, metalloprotease, the ActA protein, and the PrfA transcriptional regulator, which controls their expression as well as the expression of genes outside the island (55).

All known L. pneumophila TCSs that regulate the expression of EEGs are global regulators found in all the *Legionella* species sequenced. They are not part of genomic islands, and the signal sensed by their cognate sensor histidine kinases (SHKs) is unknown. Here, we describe a novel L. pneumophila TCS effector island. The TCS is composed of the LciS SHK, which specifically senses copper and activates the cognate LciR response regulator (RR). LciR functions as a local regulator, activating the expression of a single adjacent EEG (*lciE*). The LciRS-LciE genomic island undergoes HGT throughout the *Legionella* genus and represents a novel type of effector regulation in *Legionella*.

## RESULTS

**FIG 1:**
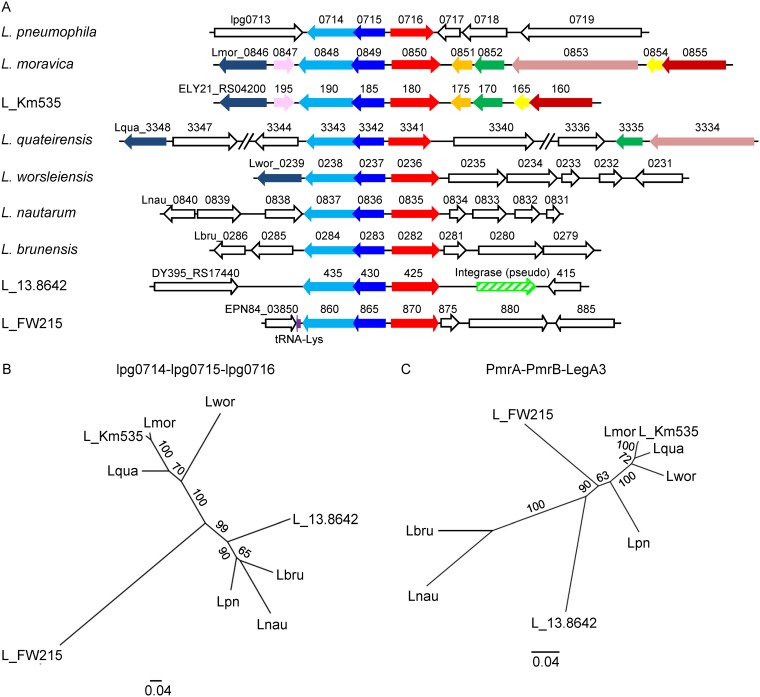
Regulator-effector island undergoes HGT in the *Legionella* genus. (A) Schematic presentation of the genes located in the genomic region near the lpg0714-lpg0715-lpg0716 orthologs in six characterized *Legionella* species, L. pneumophila (Lpg), *L. moravica* (Lmor), *L. quateirensis* (Lqua), *L. worsleiensis* (Lwor), *L. nautarum* (Lnau), and *L. brunensis* (Lbru), and three uncharacterized *Legionella* species, *Legionella* sp. strain 13.8642 (L_13.8642), *Legionella* sp. strain FW215 (L_FW215), and *Legionella* sp. strain Km535 (L_Km535), harboring the regulator-effector island. Homologous genes are marked by the same color, and nonhomologous genes are marked in white. The genes are indicated by their locus tag number. The integrase pseudogene is marked with a hatched arrow, and the Lys tRNA gene is marked by a purple arrow. (B) A maximum-likelihood phylogeny tree of nine *Legionella* species harboring the regulator-effector island reconstructed on the basis of concatenated amino acid alignment of the lpg0714-lpg0715-lpg0716 orthologous open reading frames. (C) Similarly constructed phylogeny tree of the PmrA-PmrB and LegA3 orthologous ORFs. Bootstrap values are presented on the branches.

The CpxR and PmrA direct regulators of effector-encoding genes (EEGs) belong to the winged helix-turn-helix (wHTH) family of response regulators (RRs), which function as part of the CpxRA and PmrAB two-component systems (TCSs) (18–20, 22, 23). L. pneumophila harbors a third TCS from the same family, consisting of lpg0714, which encodes a sensor-histidine kinase (SHK), and lpg0715, which encodes a wHTH-type RR. This TCS is found in L. pneumophila, in five other characterized *Legionella* species, and in three uncharacterized *Legionella* species. In all these species, a gene, which was shown in L. pneumophila to encode an effector protein (56) (lpg0716 in L. pneumophila), is located next to it, forming a regulator-effector island (Fig. 1A; see also Fig. S1 in the supplemental material).

10.1128/mBio.03232-19.1 FIG S1 Presence and absence of lpg0714-lpg0715-lpg0716 in the *Legionella* genus. A maximum likelihood tree of 41 *Legionella* species was reconstructed on the basis of concatenated amino acid alignment of 78 orthologous open reading frames (41). For each species, the presence (grey) or absence (white) of lpg0714, lpg0715, and lpg0716 genes is indicated. Download FIG S1, TIF file, 0.9 MB. Copyright © 2020 Linsky et al. 2020 Linsky et al. This content is distributed under the terms of the Creative Commons Attribution 4.0 International license.

### The lpg0714-lpg0715-lpg0716 island undergoes horizontal gene transfer in the *Legionella* genus.

Comparison of the genomic location of the regulator-effector island in the nine *Legionella* species in which it was found indicated that it is usually located in different positions (Fig. 1A). However, in genomes of the four closely related species (L. worsleiensis, L. moravica, L. quateirensis, and *Legionella* sp. strain Km535), it is positioned in a similar genomic region, which underwent additional changes in its gene content (Fig. 1A). Comparison of the genomic region of this regulator-effector island is found in L. pneumophila and in the other eight *Legionella* species revealed that it contains highly variable genes (Fig. S2A), indicating that it is prone to changes in gene content. To examine whether this regulator-effector island undergoes HGT in the *Legionella* genus, we reconstructed the phylogenetic tree of these nine species based on the protein sequences encoded by the lpg0714-lpg0715 TCS and the lpg0716 effector and compared it to the phylogenetic tree reconstructed using a TCS (PmrAB) and a core effector (LegA3) present in all the *Legionella* species. While the analysis using PmrAB-LegA3 resulted in a tree structure similar to that of the known *Legionella* phylogenetic tree (as far as the characterized *Legionella* species are concerned [41]), the tree based on the regulator-effector island resulted in a different topology (Fig. 1B). Moreover, while the GC content of the *pmrAB-legA3* DNA sequence was similar to the genomic GC content in all nine species (Fig. S2B), the GC content of the regulator-effector island in three of the species (L. pneumophila, L. brunensis, and L. nautarum) was considerably lower, suggesting a recent event of HGT in these species. The genomic position, the GC content, and the tree structure of L. worsleiensis, L. moravica, L. quateirensis, and *Legionella* sp. strain Km535 imply a single HGT event, which occurred before their speciation. Collectively, these results suggest that this regulator-effector island undergoes HGT in the *Legionella* genus as a unit.

10.1128/mBio.03232-19.2 FIG S2 Regulator-effector island undergoes HGT in the *Legionella* genus. (A) Schematic presentation of the genomic region where the lpg0714-lpg0715-lpg0716 L. pneumophila orthologs are located in the six characterized *Legionella* species, L. pneumophila (Lpn), *L. moravica* (Lmor), *L. quateirensis* (Lqua), *L. worsleiensis* (Lwor), *L. nautarum* (Lnau), and *L. brunensis* (Lbru), and the three uncharacterized *Legionella* species, *Legionella* sp. strain 13.8642 (L_13.8642), *Legionella* sp. strain FW215 (L_FW215), and *Legionella* sp. strain Km535 (L_Km535), harboring the regulator-effector island. Homologous genes are indicated by the same color, and nonhomologous genes are white. The regions between lpg0713, encoding an oligopeptide transporter, and lpg0719, encoding a valyl-tRNA synthetase in the nine species, are presented. The genes are indicated by their locus tag number. (B) Comparison of the GC content of the lpg0714-lpg0715-lpg0716 genes and the *pmrA-pmrB-legA3* genes to the genomic GC content of the nine *Legionella* species harboring the regulator-effector island. Download FIG S2, TIF file, 1.5 MB. Copyright © 2020 Linsky et al. 2020 Linsky et al. This content is distributed under the terms of the Creative Commons Attribution 4.0 International license.

### The L. pneumophila lpg0714-lpg0715 TCS is homologous to TCSs involved in copper sensing in other bacteria.

**FIG 2:**
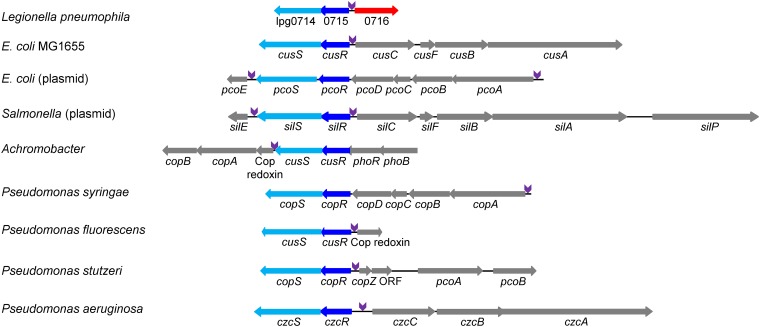
Two-component systems homologous to lpg0714-lpg0715 are present in many bacteria and are located next to genes encoding metal resistance systems. Schematic representation of genomic regions containing TCSs homologous to lpg0714-lpg0715 found in different bacteria. The homologous response regulators (RRs) are shown in dark blue, the homologous sensor histidine kinases (SHKs) are shown in light blue, the effector lpg0716 is shown in red, and the genes involved in copper resistance, or in resistance to other metals, are shown in gray (most of these genes are not homologous to one another). The position of the conserved regulatory element predicted to be recognized by the lpg0715 RR is shown in purple (Fig. S3). The different designations of the homologous RRs and SHKs are indicated.

Examination of the proteins encoded by lpg0714 and lpg0715 indicated that homologous TCSs are present in many bacteria. In most of them, the genes located next to it encode different proteins and systems involved in copper resistance (Fig. 2); however, cases of genes encoding resistance to silver and zinc were also described (57, 58). A few of these systems were studied before, including the CusRS TCS of Escherichia coli, which is located next to the CusCFBA copper transport system (59, 60). Examination of the lpg0716 EEG and of all other genes located next to the homologous TCSs from the different bacteria indicated that a conserved sequence is found at a precise distance from the predicted or validated −10 promoter elements in one or two of the surrounding genes (Fig. 2 and Fig. S3).

10.1128/mBio.03232-19.3 FIG S3 Consensus regulatory element of genes found next to the LciRS homologous TCSs in different bacteria. The regulatory region of genes located near the genes encoding the LciRS homologous TCS in different bacteria is shown: L. pneumophila (Lpg), *L. moravica* (Lmor), *L. quateirensis* (Lqua), *L. worsleiensis* (Lwor), *L. nautarum* (Lnau), *L. brunensis* (Lbru), *Legionella* sp. strain 13.8642 (L_13.8642), *Legionella* sp. strain FW215 (L_FW215), *Legionella* sp. strain Km535 (L_Km535), Escherichia coli (Esc), Salmonella enterica (Sal), Enterobacter cloacae (Ent), *Achromobacter* species (Ach), Pseudomonas aeruginosa (Pa), Pseudomonas fluorescens (Pf), Pseudomonas putida (Pp), Pseudomonas stutzeri (Ps), and Bordetella pertussis (Bper). In purple are the putative LciR inverted-repeat regulatory elements, in light blue are the two additional regulatory elements found between the inverted-repeat and the −10 promoter element, in dark blue are the −10 promoter elements, and the experimentally determined transcription start sites (TSSs) are underlined and marked in blue. Download FIG S3, TIF file, 1.5 MB. Copyright © 2020 Linsky et al. 2020 Linsky et al. This content is distributed under the terms of the Creative Commons Attribution 4.0 International license.

Due to the homology of this TCS and its predicted regulatory element to systems involved in copper sensing and detoxification, we named the L. pneumophila lpg0714-lpg0716 genes *lci*, for *Legionella* copper island. The SHK lpg0714 was named LciS, the RR lpg0715 was named LciR, and the effector lpg0716 was named LciE.

### *lciE* is induced by copper in L. pneumophila in an LciRS-dependent manner.

**FIG 3:**
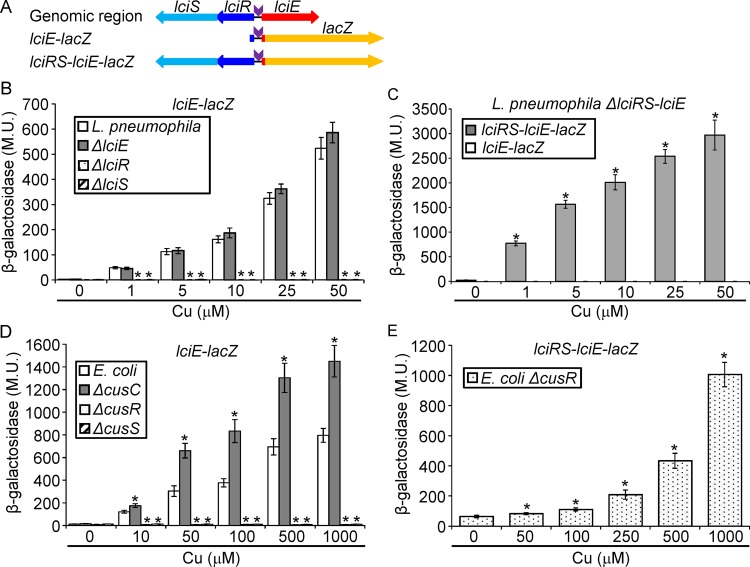
*lciE* is induced by copper in L. pneumophila and E. coli in a TCS-dependent manner. (A) Schematic representation of the L. pneumophila *lciRS-lciE* genomic region and the two *lacZ* fusions constructed (*lciE-lacZ* and *lciRS-lciE-lacZ*) to determine the expression of *lciE*. (B) The expression of the *lciE-lacZ* fusion was examined in the wild-type strain (white bars), in the *lciE* deletion mutant (gray bars), in the *lciR* deletion mutant (dotted bars), and in the *lciS* deletion mutant (hatched bars) at the stationary phase under different copper concentrations (indicated below the bars). (C) The expression of the *lciE-lacZ* fusion (white bars) and the *lciRS-lciE-lacZ* fusion (gray bars) was examined in the L. pneumophila triple *lciRS-lciE* deletion mutant at the stationary phase under different copper concentrations (indicated below the bars). (D) The expression of the *lciE-lacZ* fusion was examined in E. coli (white bars), in the E. coli *cusC* deletion mutant (gray bars), in the E. coli *cusR* deletion mutant (dotted bars), and in the *cusS* deletion mutant (hatched bars) at the stationary phase under different copper concentrations (indicated below the bars). (E) The expression of the *lciRS-lciE-lacZ* fusion was examined in the E. coli *cusR* deletion mutant at the stationary phase under different copper concentrations (indicated below the bars). The levels of expression of the *lacZ* fusions were found to be significantly different (*, *P < *10^−5^, paired Student's *t* test) between expression of the same *lacZ* fusion under the same copper concentrations between the mutants and the one in the wild-type strain (panels B and D) or between the expression of the same strain grown without copper and the one grown under different copper concentrations (panels C and E). β-Galactosidase activity was measured as described in Materials and Methods. Data (expressed in Miller units [M.U.]) are the averages ± standard deviations (error bars) of the results from at least three different experiments.

To determine whether *lciE* is induced in response to copper exposure, two fusions were constructed (Fig. 3A). The first fusion contains the 300-bp regulatory region of *lciE* fused to *lacZ* (designated *lciE-lacZ*). The second construct contains the same fusion as well as the *lciRS* genes in their original genomic organization (designated *lciRS-lciE-lacZ*) (Fig. 3A). To examine the expression of these constructs in response to copper exposure, we first determined the concentrations of copper that L. pneumophila can tolerate (Fig. S4A) and examined the effect of copper on the expression of *lciE* using a range of concentrations, from 1 to 50 μM. Using the *lciE-lacZ* fusion, the level of expression of *lciE* increased gradually as the concentration of copper was elevated, reaching more than 100-fold induction at the highest copper concentration (Fig. 3B). To determine the importance of the LciRS TCS for LciE expression, four deletion mutants were constructed, *lciR*, *lciS*, and *lciE* single deletion mutants and an *lciRS-lciE* triple deletion mutant. None of these mutants had an intracellular growth phenotype when examined in the amoeba host Acanthamoeba castellanii and in HL-60-derived human macrophages, as well as in a competition assay in amoeba with and without the addition of copper (Fig. S5). The copper induction of *lciE* was found to be completely dependent on the *lciR* and *lciS* genes, and as expected, the *lciE* deletion did not affect the levels of expression of the *lciE-lacZ* fusion (Fig. 3B). To further substantiate these results, copper induction was examined in an L. pneumophila strain deleted for the entire *lciRS-lciE* region (Fig. 3C). Using the *lciE-lacZ* fusion (Fig. 3A), no induction by copper was obtained, but when the *lciRS-lciE-lacZ* fusion (Fig. 3A) was examined, high levels of expression were obtained due to exposure to copper, which increased gradually as the concentrations of copper increased (Fig. 3C). In addition, the expression of *lciE* was found to be completely dependent on the presence of a functional LciRS TCS, since no induction was obtained when the conserved histidine of the LciS SHK and conserved aspartic acid of the LciR RR were mutated (Fig. S6A). Collectively, these results indicate that the expression of the Icm/Dot effector LciE is activated by copper, and its activation is completely dependent on the presence and functionality of the LciRS TCS.

10.1128/mBio.03232-19.4 FIG S4 Effect of copper on L. pneumophila and E. coli growth in media. L. pneumophila (A) and E. coli (B) were grown with different concentrations of copper (indicated on the right), and their growth (OD_600_) was examined in intervals of 1 h for the time indicated. Download FIG S4, TIF file, 1.7 MB. Copyright © 2020 Linsky et al. 2020 Linsky et al. This content is distributed under the terms of the Creative Commons Attribution 4.0 International license.

10.1128/mBio.03232-19.5 FIG S5 LciRS TCS and the effector LciE are dispensable for intracellular growth. (A and B) The intracellular growth of the *lciR*, *lciS*, *lciE*, and *lciRS-lciE* deletion mutants was examined in A. castellanii (A) and HL-60-derived human macrophages (B). Symbols: diamonds, L. pneumophila wild-type strain JR32; squares, *icmT* mutant; triangles, *lciS* deletion mutant; black circles, *lciR* deletion mutant; star, *lciE* deletion mutant; white circles, *lciRS-lciE* triple deletion mutant. The experiments were performed three times, and similar results were obtained. (C and D) Intracellular competition assay between the L. pneumophila *lciRS-lciE* triple deletion mutant (white circles) and the JR32 wild-type strain (diamonds) in A. castellanii. The experiment was performed without (C) and with (D) 200 μM copper. Download FIG S5, TIF file, 0.8 MB. Copyright © 2020 Linsky et al. 2020 Linsky et al. This content is distributed under the terms of the Creative Commons Attribution 4.0 International license.

10.1128/mBio.03232-19.6 FIG S6 Effect of mutations and copper on the expression of *lciE* and *lciR*. (A) The effect of mutations on the amino acids predicted to be required for phosphorylation of LciR (LciR^D51A^) and LciS (LciS^H148A^) on *lciE* induction by copper. The levels of expression of wild-type *lciRS-lciE-lacZ* fusion and the *lciRS-lciE-lacZ* fusion containing a mutation in the amino acids, with and without 50 μM copper, are indicated below the bars. The levels of expression of the *lacZ* fusions were found to be significantly different (*, *P < *10^−5^, paired Student’s *t* test) between mutated fusions and the wild-type fusion after exposure to copper. (B) *lciR* expression is activated neither by copper nor by the LciR regulator. The expression of the *lciR-lacZ* fusion was examined in the wild-type strain and in the *lciR* deletion mutant with (grey bars) and without (white bars) the addition of 50 μM copper. Download FIG S6, TIF file, 0.7 MB. Copyright © 2020 Linsky et al. 2020 Linsky et al. This content is distributed under the terms of the Creative Commons Attribution 4.0 International license.

### *lciE* is induced in E. coli after copper exposure in a CusRS- or LciRS-dependent manner.

As indicated above, the LciRS homologous TCSs as well as the LciR predicted regulatory element are conserved in many bacteria. To examine if these TCSs function similarly, we examined the expression of the *lciE-lacZ* construct (Fig. 3A) in E. coli. To this end, we determined the maximal copper concentration that E. coli tolerates without any effect on growth to be 1 mM (Fig. S4B), which is much higher than the one found for L. pneumophila (Fig. S4A). Analysis of the *lciE-lacZ* construct in E. coli resulted in gradual induction of *lciE* expression as the concentration of copper increased (Fig. 3D). Moreover, this induction was completely dependent on the E. coli CusRS TCS (Fig. 3D). Interestingly, when *lciE-lacZ* expression was examined in the *cusC* deletion mutant (CusC together with CusFBA form a copper transporter located in the E. coli envelope [59, 61]), higher levels of expression of *lciE* were observed at all copper concentrations examined (Fig. 3D). This result was obtained probably because E. coli lacking a functional CusCFBA system is exposed to higher concentrations of copper, which led to higher expression of *lciE-lacZ*. Comparing this result to the one obtained with the L. pneumophila *lciE* deletion mutant (compare Fig. 3B and D), which was induced similarly to wild-type L. pneumophila at all copper concentrations, indicates that in L. pneumophila, LciE is not involved in copper transport when the bacteria are grown in media, as expected from an Icm/Dot effector protein that functions during infection of host cells. Furthermore, the higher concentrations of copper tolerated by E. coli led us to examine the copper induction of the L. pneumophila LciRS TCS using E. coli as an *in vivo* heterologous system. Examination of the *lciRS-lciE-lacZ* construct in an E. coli *cusR* deletion mutant indicated that the L. pneumophila LciRS functions in E. coli and can respond to higher concentrations of copper than those tolerated by L. pneumophila (Fig. 3E). Collectively, these results indicate that the CusRS and LciRS TCSs function similarly, and both activate the expression of *lciE* in response to copper exposure.

### LciR recognizes a conserved regulatory element located upstream of *lciE*.

**FIG 4:**
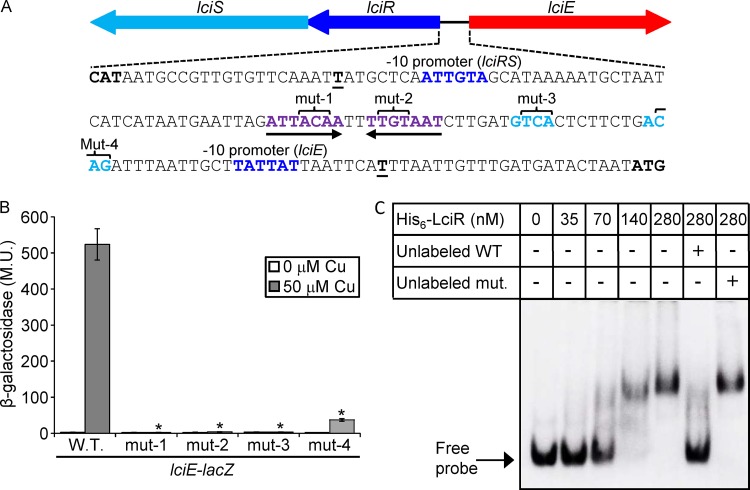
LciR directly regulates the expression of *lciE* using a conserved regulatory element. (A) The intergenic DNA sequence located between *lciR* and *lciE*. The *lciR* and *lciE* −10 promoter elements are in dark blue, and the nucleotides representing the putative LciR consensus are in purple (the inverted-repeat sequence) or light blue (the two sequences located between the inverted repeat and the *lciE* −10 promoter); the inverted repeat is also marked with arrows. The transcription start sites are boldface and underlined. The four triplets of base pairs mutated in each part of the suspected regulatory element are indicated. (B) The effect of mutations in the putative LciR regulatory element. The levels of expression of wild-type *lciE-lacZ* fusion and the *lciE-lacZ* fusions containing mutations in the nucleotides marked in panel A were examined with and without 50 μM copper. The levels of expression of the *lacZ* fusions were found to be significantly different (*, *P < *10^−5^, paired Student's *t* test) between the fusions containing the wild-type regulatory region and the mutated regulatory region under the same copper concentrations. β-Galactosidase activity was measured as described in Materials and Methods. Data (expressed in Miller units [M.U.]) are the averages ± standard deviations (error bars) of the results from at least three different experiments. (C) L. pneumophila His_6_-LciR protein binds to the *lciE* regulatory region. Gel mobility shift assay was performed with purified His_6_-LciR protein and the DIG-labeled *lciE* regulatory region. The first lane did not contain any protein. The rest of the lanes contained increasing amounts of the His_6_-LciR protein in 2-fold increments, starting from 35 nM. Competition was performed using unlabeled probe as a specific competitor (unlabeled WT) or a probe containing a mutation in the LciR regulatory element (unlabeled mut.).

We identified a conserved regulatory element located upstream of *lciE* and other genes known or expected to be regulated by LciRS homologous TCSs (Fig. 2 and Fig. S3). This regulatory element constitutes a 7-bp inverted repeat (ATTACAAnnTTGTAAT) as well as two shorter (4 bp each) conserved sequences located between the inverted repeat and the *lciE* −10 promoter element (Fig. 4A and Fig. S3). Examination of the L. pneumophila genomic sequence for the presence of additional such regulatory elements revealed that this site is not present elsewhere in the genome, which is in line with our observation that the LciRS-LciE genomic island was acquired by HGT. To determine the importance of each of the putative *lciE* regulatory elements for the copper induction by LciR, they were mutagenized and examined for their levels of expression before and after copper induction. Three of these mutated *lciE-lacZ* fusions completely lost their ability to be induced by copper, and the fourth mutant retained a very limited ability to respond to copper (Fig. 4B).

Since the *lciR* gene that encodes the RR and the *lciE* EEG share an intergenic region (Fig. 4A), we examined whether the *lciR* gene is activated by copper and whether it is an autoregulator. The *lciR* gene was neither induced by copper nor affected by the *lciR* deletion mutant (Fig. S6B). These results indicate that the LciRS TCS specifically activates the expression of *lciE* in response to copper and suggest that the two conserved short regulatory elements located between the inverted repeat and the −10 promoter element of *lciE* play a critical role in directing the activation by LciR to *lciE* and not to *lciR*.

### The L. pneumophila LciR protein directly binds to the regulatory region of *lciE*.

To further support the results presented, the L. pneumophila LciR protein was His tagged, overexpressed, purified, and used for gel mobility shift assays with the *lciE* regulatory region. The L. pneumophila His_6_-LciR protein was found to bind to the regulatory region of the *lciE* gene, as evidenced by a shift in the migration of the DNA probe (Fig. 4C). The band shift degree as well as the amount of the shifted probe correlated with the increasing amounts of His_6_-LciR (Fig. 4C). In addition, competition with an unlabeled probe reduced the band shift (Fig. 4C, compare lanes 5 and 6). To further validate the specificity of the binding, we performed additional competition assays with an unlabeled probe containing a mutation in the LciR binding site. When the unlabeled mutated probe was used, a dramatic decrease in competition was observed compared to that of the unlabeled wild-type probe (Fig. 4C, compare lanes 6 and 7).

The mobility shift assay (Fig. 4C), together with the examination of *lciE* gene expression in the *lciR* and *lciS* deletion mutants (Fig. 3) and the analysis of the mutations in the LciR consensus sequence (Fig. 4B), establish LciR as a direct regulator of the *lciE* EEG in L. pneumophila.

### Identification of amino acids required for LciS copper sensing.

**FIG 5:**
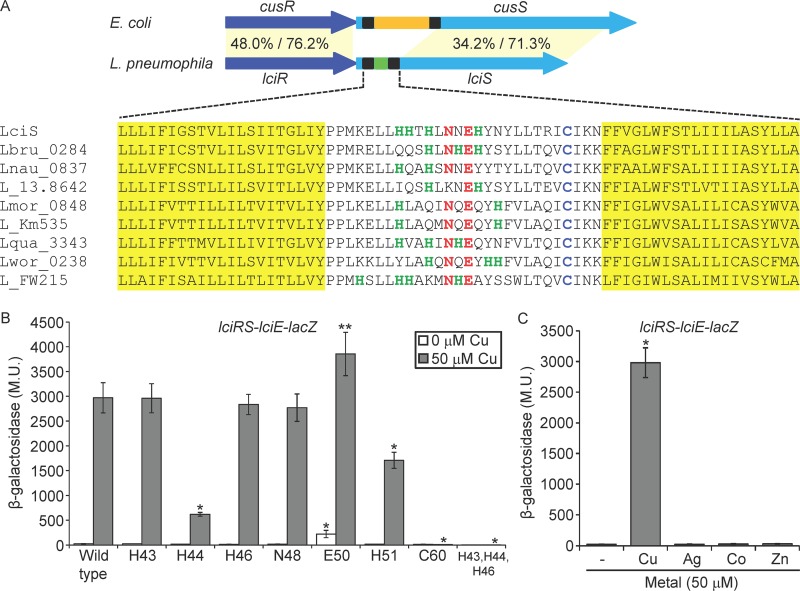
Identification of amino acids required for copper induction located in the LciS periplasmic sensor domain. (A) Schematic illustration of the L. pneumophila *lciRS* and the E. coli *cusRS* genes. The periplasmic domain of the nine *Legionella* SHKs is much smaller than the periplasmic domain found in other bacteria, such as E. coli (28 amino acids compared to about 150 amino acids, respectively). In addition, protein sequence alignment of the LciS transmembrane regions and periplasmic domains from nine *Legionella* species harboring the LciRS TCS is shown. The amino acids that compose the SHK predicted transmembrane domains are colored yellow. Amino acids found in the periplasmic domain which might be involved in copper-binding are marked by different colors: histidine residues, green; cysteine, light blue; and asparagine and glutamic acid, red. (B) The effect of mutations in the periplasmic domain of the L. pneumophila LciS on copper induction. The levels of expression of wild-type *lciRS-lciE-lacZ* fusion and the *lciRS-lciE-lacZ* fusions containing mutations in the amino acids indicated below the bars, with and without 50 μM copper. The levels of expression of the *lacZ* fusions were found to be significantly different (**, *P < *10^−4^; *, *P < *10^−5^; both by paired Student's *t* test) between the mutated fusions and the wild-type fusion with or without copper. (C) The effect of different metals on *lciE* expression. The levels of expression of wild-type *lciRS-lciE-lacZ* fusion after exposure to the metals indicated below the bars (50 μM). The level of expression of the *lacZ* fusion was found to be significantly different (*P < *10^−5^, paired Student's *t* test) between expression with metals and the one without metal exposure. β-Galactosidase activity was measured as described in Materials and Methods. Data (expressed in Miller units [M.U.]) are the averages ± standard deviations (error bars) of the results from at least three different experiments.

Even though the E. coli CusRS and the L. pneumophila LciRS TCSs are homologous and both respond to elevated copper concentrations (Fig. 3), the periplasmic sensing domain of CusS and LciS are considerably different in size (150 amino acids in E. coli compared to 28 amino acids in L. pneumophila) and show no sequence homology (Fig. 5A). To identify amino acids required for copper induction in the L. pneumophila LciS periplasmic sensor domain, we aligned the sequences of LciS from the nine *Legionella* species harboring it. Since the amino acids histidine, cysteine, and methionine were previously shown to be involved in copper binding (62, 63), we specifically looked for these residues. Several histidine residues and a single cysteine were found in the short LciS periplasmic domain, together with other conserved amino acids (Fig. 5A). To examine the importance of these conserved amino acids for copper sensing by LciS, seven amino acid residues in the periplasmic domain of LciS (H43, H44, H46, N48, E50, H51, and C60) were changed separately to alanine residues. These mutations were introduced into the *lciRS-lciE-lacZ* fusion and used to determine the levels of copper induction (Fig. 5B). The most striking result was obtained with the mutation of the cysteine at position 60, which completely abolished the induction by copper. In addition, two of the histidine residues mutated (H44 and H51) significantly reduced induction by copper. We noticed that the number of histidine residues varies between the periplasmic sensor domains of the different *Legionella* LciS proteins, but at least two histidine residues were present in each LciS periplasmic domain. Thus, we constructed a triple mutation in which the three adjacent histidine residues (H43, H44, and H46) all were changed to alanine residues. This combined mutation resulted in a complete lack of induction by copper (Fig. 5B). To determine the specificity of LciS, we also exposed the bacteria to other similar metals (57, 64), and as seen in Fig. 5C, the LciRS TCS was found to be specific to copper and no induction was obtained with any of the other metals examined. Collectively, these results indicate that the *Legionella* LciS small periplasmic domain contains few histidine residues and a single cysteine residue specifically required for copper sensing.

### The LciRS-LciE genomic island is regulated by the Fis repressors.

**FIG 6:**
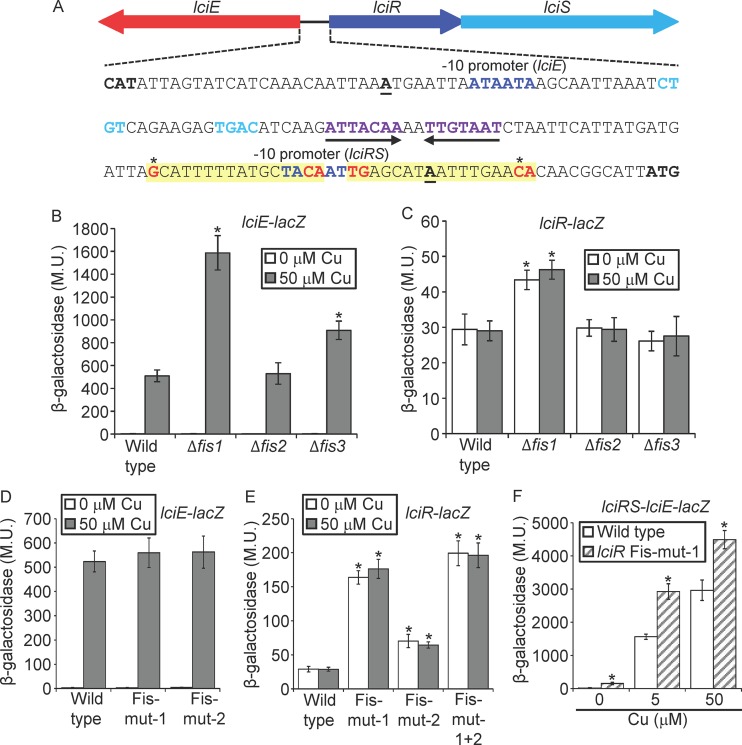
*lciRS-lciE* island is repressed by Fis. (A) The intergenic DNA sequence located between *lciE* and *lciR*. The *lciR* and *lciE* −10 promoter elements are in dark blue, and the nucleotides representing the LciR consensus are in purple (the inverted-repeat sequence) or light blue (the two sequences located between the inverted repeat and the −10 promoter of *lciE*); the inverted repeat is also marked with arrows. The transcription start sites are boldface and underlined. The putative *lciR* Fis regulatory elements are shaded in yellow, conserved nucleotides of the Fis consensus are marked in red, and the nucleotides mutated are marked by asterisks. (B and C) The expression levels of the *lciE-lacZ* fusion (B) and the *lciR-lacZ* fusion (C) were examined in the wild-type strain and in the three *fis* deletion mutants at the stationary phase. Expression was examined with (gray bars) and without (white bars) 50 μM copper. The levels of expression of the *lacZ* fusions were found to be significantly different (*, *P < *10^−5^, paired Student's *t* test) between expression of the wild-type strain and each *fis* deletion mutant under the same conditions. (D and E) The levels of expression of wild-type *lciE-lacZ* fusion and the two *lciE-lacZ* fusions containing mutations (mut-1 and mut-2) in the putative Fis regulatory elements (D) and wild-type *lciR-lacZ* fusion and the three *lciR-lacZ* fusions containing mutations (mut-1, mut-2, and mut-1 + 2) in the putative Fis regulatory elements (E) were examined with (gray bars) and without (white bars) 50 μM copper. The levels of expression of the *lacZ* fusions were found to be significantly different (*, *P < *10^−5^, paired Student's *t* test) between fusions containing the wild-type regulatory region and the mutated regulatory region under the same copper concentrations. (F) The levels of expression of wild-type *lciRS-lciE-lacZ* fusion (white bars) and the same fusion containing a mutation in the downstream Fis regulatory element of *lciR* (hatched gray bars) were examined in the *lciRS-lciE* deletion mutant without copper and with 5 μM and 50 μM copper. The levels of expression of the *lacZ* fusions were found to be significantly different (*, *P < *10^−5^, paired Student's *t* test) between the wild-type fusion and the mutated fusion under the same copper concentrations. β-Galactosidase activity was measured as described in Materials and Methods. Data (expressed in Miller units [M.U.]) are the averages ± standard deviations (error bars) of the results from at least three different experiments.

It was previously shown in S. enterica as well as in other bacteria that genomic islands that undergo HGT are silenced by nucleoid-associated proteins (NAPs), such as H-NS and Hha (65–67). Since the LciRS-LciE island undergoes HGT in the *Legionella* genus (Fig. 1 and Fig. S1 and S2), we were interested in examining whether this island is silenced by NAPs. The H-NS NAP is not present in *Legionella*, but three Fis paralogs (Fis1, Fis2, and Fis3), which are also NAPs, were previously shown to directly repress the expression of EEGs (32). Examination of the *lciRS-lciE* intergenic region led to the identification of four potential Fis regulatory elements (TG-N_13_-C), two close to or overlapping the −10 promoter element of *lciE* (Fig. S7) and two others close to or overlapping the −10 promoter element of *lciR* (Fig. 6A and Fig. S7). To determine whether the Fis repressors are involved in the regulation of the LciRS-LciE island, the expression of the *lciE-lacZ* fusion was examined in deletion mutants of each of the three *fis* genes. In the absence of copper, the expression levels of the *lciE-lacZ* fusion in the three *fis* deletion mutants were similar to those in the wild-type strain (Fig. 6B). However, in the presence of copper, the expression of the *lciE-lacZ* fusion was significantly higher in the *fis1* and *fis3* deletion mutants (Fig. 6B). In addition, when the expression of the *lciR-lacZ* fusion was examined in the same mutants under the same conditions, there was an increase in the level of expression of the *lciR-lacZ* fusion in the *fis1* deletion mutant that was independent of the presence of copper (Fig. 6C). These results indicate a direct repression of Fis on *lciE*, on *lciR*, or on both genes.

10.1128/mBio.03232-19.7 FIG S7 Predicted Fis regulatory elements in the regulatory regions of *lciE* and *lciR* homologs in different *Legionella* species. The promoter region of the *lciE* (A) and *lciR* (B) genes in the nine *Legionella* species harboring the island, L. pneumophila (*lciE* and *lciR*), *L. moravica* (Lmor), *L. quateirensis* (Lqua), *L. worsleiensis* (Lwor), *L. nautarum* (Lnau), *L. brunensis* (Lbru), *Legionella* sp. strain 13.8642 (L_13.8642), *Legionella* sp. strain FW215 (L_FW215), and *Legionella* sp. strain Km535 (L_Km535). In purple are the putative LciR inverted-repeat regulatory elements, in light blue are the two additional regulatory elements found between the inverted repeat and the −10 promoter element of *lciE*, in dark blue are the −10 promoter elements, and the experimentally determined transcription start sites (TSSs) are underlined and marked in blue. The predicted Fis sites in both promoter regions are marked: the site overlapping or close to the −10 promoter element is colored yellow, and a second predicted Fis site (when present) is underlined. In both predicted Fis sites, the conserved TG and CA consensus nucleotides are in red and the nucleotides mutated are marked by asterisks. Download FIG S7, TIF file, 1.8 MB. Copyright © 2020 Linsky et al. 2020 Linsky et al. This content is distributed under the terms of the Creative Commons Attribution 4.0 International license.

### Fis1 and Fis3 repress the expression of *lciR* and affect the copper induction of *lciE*.

To distinguish between the three possibilities described above, we constructed site-directed mutations in the four described putative Fis regulatory elements (Fig. 6A and Fig. S7). The two mutations constructed in the putative Fis regulatory elements of *lciE* did not affect its level of expression, with or without copper (Fig. 6D). However, both mutations constructed in the putative Fis regulatory element of *lciR* (Fig. 6A) showed a relief of repression (Fig. 6E). The mutation in the Fis regulatory element located immediately downstream to the −10 promoter element of *lciR* (Fig. 6A) showed a 6-fold relief of repression, and the mutation in the upstream Fis site showed 2-fold relief of repression. A combined mutation in both Fis regulatory elements led to a level of expression similar to that of the mutation in the downstream Fis site (Fig. 6E). It is important to note that the Fis regulatory element located downstream to the *lciR* −10 promoter element is conserved in all nine *Legionella* species harboring the LciRS-LciE genomic island, while the three other putative Fis regulatory elements examined are not conserved (Fig. S7). The effect observed on the level of expression of the *lciR-lacZ* fusion containing the mutations in the Fis regulatory elements was considerably stronger than the effect observed on the wild-type *lciR-lacZ* fusion in the *fis1* deletion mutant. Since a double deletion of both *fis1* and *fis3* is not viable in L. pneumophila (32), only the mutation in the Fis regulatory element can fully expose the degree of repression mediated by both Fis1 and Fis3 on the expression of LciR. To determine the effect of the Fis repression of LciR on the expression of LciE, we used the *lciRS-lciE-lacZ* fusion to construct a single-base-pair mutation in the downstream Fis regulatory element of LciR and examined the expression of LciE from this fusion. As can be seen in Fig. 6F, the level of expression of the *lciRS-lciE-lacZ* fusion containing the mutation in the *lciR* regulatory element was much higher in the absence of copper (6-fold) as well as in the presence of copper compared to that of the wild-type fusion. Collectively, these results indicate that the repression mediated by Fis on the LciR regulator affects the level of expression of the LciE effector with and without copper, showing that Fis proteins silence this genomic island by repressing the expression of the positive regulator LciR.

## DISCUSSION

**FIG 7:**
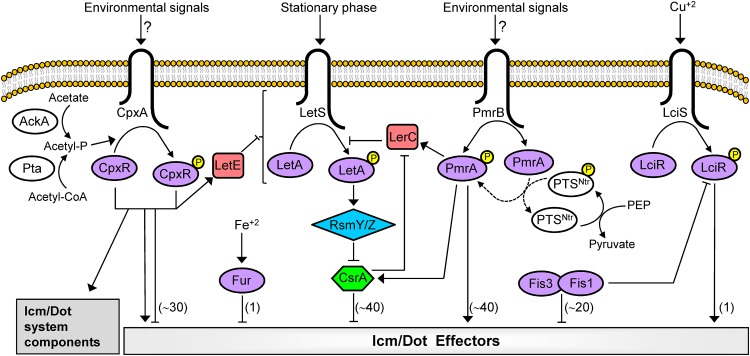
Model of the regulatory network of direct regulators that regulate the expression of L. pneumophila EEGs. The four TCSs, CpxRA, PmrAB, LciRS, and LetAS, as well as the components of the LetAS-RsmYZ-CsrA regulatory cascade, which were found to be involved in the regulation of EEGs, are shown. The four SHKs (CpxA, LetS, PmrB, and LciS) are drawn inside the bacterial inner membrane. The four RRs (CpxR, LetA, PmrA, and LciR), as well as the other DNA binding regulators (Fur, Fis1, and Fis3), are colored purple. The RRs are phosphorylated (small yellow circle) and/or dephosphorylated by their cognate SHKs. The CsrA RNA binding regulator is colored green, and the RsmY/RsmZ small RNAs are colored blue. Connector proteins that participate in the regulation of EEGs are colored red, and modulators are colored white. Acetyl-P, acetyl phosphate; PEP, phosphoenolpyruvate; PTS, phosphotransferase system. The numbers of EEGs that were shown to be regulated by each of the regulatory systems are indicated in parentheses. Arrows and T-shaped symbols indicate activation and repression, respectively. Solid lines indicate direct regulation and broken lines indirect regulation.

The L. pneumophila Icm/Dot secretion system translocates into host cells the largest number of effectors known in a single bacterium, and these effectors manipulate numerous host cell processes for the benefit of the bacteria (10–14). One of the challenges encountered by bacteria using such a multicomponent pathogenesis system is the coordination of the expression of the pathogenesis genes encoding these components to result in a successful infection. Thus far, several regulatory systems that control the expression of EEGs were identified in L. pneumophila (Fig. 7), including a pair of TCSs (PmrAB and CpxRA) that belong to the wHTH family of transcriptional regulators (68). Here, we described a third L. pneumophila TCS (LciRS) that belongs to the wHTH family of RRs, which directly regulates the expression of a single EEG (Fig. 7). In contrast to PmrA and CpxR, LciR is present only in several *Legionella* species and undergoes HGT as part of a genomic island together with the single EEG it regulates, representing the first case of local regulation of Icm/Dot effectors in *Legionella*.

One of the most interesting findings regarding the LciRS-LciE genomic island is that it undergoes HGT in the *Legionella* genus (Fig. 1; see also Fig. S1 and S2 in the supplemental material). Two aspects regarding this genomic island were left unresolved, namely, (i) the way by which it is transferred between *Legionella* species and (ii) the way by which it integrates into the bacterial genome. A hint regarding these two issues was obtained when we analyzed the LciRS-LciE genomic island found in two uncharacterized *Legionella* species (*Legionella* sp. strain 13.8642 and *Legionella* sp. strain FW215). In *Legionella* sp. strain 13.8642, a pseudogene was found to be located next to the LciRS-LciE genomic island (Fig. 1). This pseudogene contains a deletion of a single nucleotide after nucleotide 41, making the protein it used to encode nonfunctional. However, prior to its pseudogenization, this gene encoded a protein with a high degree of homology to a phage integrase. Homologous integrase-encoding genes were found intact in several *Legionella* species (but not next to the LciRS-LciE genomic island) and in *Legionella* sp. strain 13.8642 it is completely intact, excluding the single-nucleotide deletion described above. It is possible that this integrase was involved in the integration of the LciRS-LciE genomic island in this *Legionella* species. Integrase-encoding genes, as well as mutated integrase-encoding genes, were previously shown to be located near genomic islands in other bacteria (69, 70). Another known feature of genomic islands is that they sometimes integrate into bacterial genomes next to tRNA genes (50, 71). In this case too, in one of the uncharacterized *Legionella* species, *Legionella* sp. strain FW215, the LciRS-LciE genomic island was found in proximity to a Lys tRNA gene (Fig. 1). This indicates that tRNA genes are also an entry site for the LciRS-LciE genomic island in *Legionella*. Although the precise mechanism by which the LciRS-LciE genomic island undergoes HGT in bacteria is not known, the above-mentioned findings suggest that it utilizes transfer and integration mechanisms similar to the ones previously described for genomic islands in other bacteria.

The LciRS TCS was found to be activated in the presence of copper (Fig. 3), and homologous TCSs present in other bacteria are also activated by copper and are usually located next to genes involved in metal resistance (mainly copper) (59). Comparison of the *Legionella* LciRS TCS to the homolog TCSs present in other bacteria resulted in the identification of a major difference: the *Legionella* SHK LciS was found to contain a small (28 amino acids long) (Fig. 5A) periplasmic sensor domain, which is completely nonhomologous to the periplasmic sensing domains found in the homologous SHKs present in other bacteria, including the ones that sense copper. This finding is intriguing, since SHKs were previously shown to change their sensing domain by recombination or mutations and, in this way, change the signal they respond to and deviate from other SHKs (72). However, in the case of LciS, the sensing domain was completely changed but the signal recognized by the SHK remained the same. One can speculate on the evolutionary driving forces that can lead a sensing domain to be replaced without changing the signal it responds to. For example, an advantage can be achieved if the new sensing domain alters the sensitivity or specificity to the signal. However, examination of these two aspects, by comparing the E. coli CusS and L. pneumophila LciS SHKs, did not result in differences in the sensitivity to copper (compare Fig. 3B and D) or in the specificity to copper (Fig. 5C and data not shown). Even though the LciS periplasmic sensor domain is not homologous to other sensing domains that sense copper, it is important to mention that we did recognize specific amino acids critical for the sensing of copper by the L. pneumophila LciS, and the same amino acids were previously shown to be involved in copper binding in different proteins (62, 63).

Genes regulated by regulatory systems that sense a specific signal were shown to encode proteins with functions directly related to the signal sensed by their regulators. This phenomenon was demonstrated in many systems, including regulators of amino acids biosynthetic pathways and regulators of metal resistance systems (59, 73). In addition, the L. pneumophila *mavN* EEG, which encodes an iron transporter localized to the LCV, is regulated by the iron-specific repressor Fur (33, 34). However, in many cases the signal sensed by a regulator is not directly related to the function mediated by the genes it regulates. In these cases, the signal sensed indicates a change in the bacterial environment. Such cases were described in many pathogenesis systems, such as the S. enterica PhoPQ TCS, which senses Mg^2+^ and cationic antimicrobial peptides (74, 75), and the S. enterica PmrAB TCS, which senses acidic pH and high Fe^3+^ concentrations (76, 77). Both of these TCSs regulate the expression of numerous genes encoding diverse functions (78, 79). The function of LciE, which is activated by the LciRS TCS in response to copper, is currently unknown, and it contains no known protein domains. However, we identified two other L. pneumophila effectors with unknown functions that show a significant degree of homology to LciE (Fig. S8). The core effector CetLp1 (lpg0140), and another effector (lpg2888) that is found in most of the *Legionella* species examined, harbor a protein domain homologous to LciE (Fig. S8). In addition, these three effectors contain four predicted transmembrane domains located at the C-terminal half of the protein (Fig. S8). These similarities among the three effectors suggest that they perform a related function. Since both copper and zinc are used by eukaryotic cells to kill invading bacteria (80, 81), we examined the copper- or zinc-dependent induction of CetLp1 and lpg2888, or an effect of LciR on their levels of expression, but their expression was unaffected (Fig. S9A and B). Moreover, we generated a triple deletion mutant of *cetLp1*, lpg288, and *lciE* and examined this mutant using a competition assay in amoeba with and without the addition of copper, and no intracellular growth phenotype was observed (Fig. S9C to F).

10.1128/mBio.03232-19.8 FIG S8 L. pneumophila contains two effectors with a domain homologous to LciE. Schematic representation of the effectors LciE (lpg0716), CetLp1 (lpg0140), and lpg2888. The domain homologous among the three effectors is marked in light blue, and putative transmembrane domains are marked in black. In addition, protein sequence alignment of the homologous domain found in the three effectors is shown from the six characterized *Legionella* species in which the LciRS-LciE genomic island was found, L. pneumophila (LciE/lpg2888/CetLp1), *L. moravica* (Lmor), *L. quateirensis* (Lqua), *L. worsleiensis* (Lwor), *L. nautarum* (Lnau), and *L. brunensis* (Lbru). Download FIG S8, TIF file, 2.1 MB. Copyright © 2020 Linsky et al. 2020 Linsky et al. This content is distributed under the terms of the Creative Commons Attribution 4.0 International license.

10.1128/mBio.03232-19.9 FIG S9 Examination of the connection of CetLp1 and lpg2888 to copper and LciE. The levels of expression of *cetLp1* (A) and lpg2888 (B) *lacZ* fusions were examined without the addition of metals and after exposure to 50 μM copper (grey bars) or zinc (hatched bars). The same fusions were also examined in the *lciR* deletion mutant under the same conditions. (C and D) Intracellular competition assay between the L. pneumophila *cetLp1*-lpg2888 double deletion mutant (circles) and the JR32 wild-type strain (diamonds) in A. castellanii. (E and F) Intracellular competition assay between the L. pneumophila *cetLp1*-lpg2888-*lciE* triple deletion mutant (circles) and the JR32 wild-type strain (diamonds) in A. castellanii. The experiment was performed without (C and E) and with (D and F) 200 μM copper. Download FIG S9, TIF file, 1.5 MB. Copyright © 2020 Linsky et al. 2020 Linsky et al. This content is distributed under the terms of the Creative Commons Attribution 4.0 International license.

Unlike all the regulatory systems described thus far for L. pneumophila effectors, the LciRS-LciE genomic island represents a new type of effector regulation, unprecedented in the *Legionella* genus. In this system, a single effector is locally regulated by a dedicated regulatory system in response to a specific signal. Moreover, both the regulatory system and the effector form a unit that undergoes HGT in the *Legionella* genus. Uncovering this novel type of regulation sheds new light on the ways the effector repertoire of *Legionella* evolves and the activating signals of effectors expand.

## MATERIALS AND METHODS

### Bacteria strains and media.

The L. pneumophila wild-type strain used in this work was JR32, a streptomycin-resistant, restriction-negative mutant of L. pneumophila Philadelphia-1, which is a wild-type strain in terms of intracellular growth (82). In addition, mutant strains derived from JR32 that were used in this study are listed in Data Set S1 in the supplemental material. The E. coli strains used in this work are also listed in Data Set S1. Bacterial media, plates, and antibiotics were as previously described (83).

10.1128/mBio.03232-19.10 DATA SET S1 Strains, plasmids, and primers used in this study. Download Data Set S1, XLSX file, 0.03 MB. Copyright © 2020 Linsky et al. 2020 Linsky et al. This content is distributed under the terms of the Creative Commons Attribution 4.0 International license.

### Plasmid construction.

To construct *lacZ* translational fusions (Data Set S1), the 300-bp regulatory regions of the *lciE* and *lciR* genes were amplified by PCR using the primers listed in Data Set S1. The PCR products were then digested with BamHI and EcoRI, cloned into pGS-lac-02, and sequenced. The *lciE lacZ* fusion, which does not contain the *lciRS* genes, was designated *lciE-lacZ*. In addition, a second *lciE lacZ* fusion was constructed containing the *lciRS* genes in an organization similar to that in the genome. To construct this fusion, an internal EcoRI site present in the *lciR* gene was mutagenized in a way that does not change the LciR amino acid sequence. A 1,950-bp region was amplified by PCR using the four primers listed in Data Set S1, digested with BamHI and EcoRI cloned into pGS-lac-02, and sequenced. This *lciE lacZ* fusion, which contains the *lciRS* genes, was designated *lciRS-lciE-lacZ*.

To construct a substitution mutation in the putative LciR binding site in the regulatory region of the *lciE* gene, a substitution mutation in the putative Fis binding site in the regulatory region of the *lciE* and *lciR* genes, and substitution mutations in the *lciR* and *lciS* coding sequence, site-directed mutagenesis was performed by regular PCR or the PCR overlap extension approach (84), as previously described (19). The primers used for the mutagenesis are listed in Data Set S1, and the plasmids resulting from the site-directed mutagenesis are listed in Data Set S1.

To construct deletion substitution mutants in the L. pneumophila *lciE* and *lciS* genes, a 1-kb DNA fragment located on each side of the planned deletion was amplified by PCR using the primers listed in Data Set S1. The resulting plasmids were digested with the suitable enzymes, and the inserts were used for a four-way ligation containing the Km resistance cassette (Pharmacia). The plasmids generated, pAA-lpg0714-Km and pMLpUC18+0716Up-Km-Dw (Data Set S1), were digested with PvuII, and the resulting fragment was cloned into the pLAW344 allelic exchange vector digested with EcoRV to generate the plasmids pAA-lpg0714-pLAW and pMLpLAW344-0716-Up-Km-Dw (Data Set S1). In addition, the insert of pMLpUC18+0716Up-Km-Dw was also cloned into the pGY100 allelic exchange vector, digested with XmnI, to generate the plasmid pMLpGY100+lpg0716-Up-Km-Dw, which was later digested with SalI to take out the Km resistance cassette and generate the plasmid pMLpGY100+lpg0176-UP+Dw (Data Set S1). The latter plasmid was used to generate a clean *lciE* deletion.

To generate the triple *lciRS-lciE* deletion mutant, the insert containing the 1-kb upstream region of *lciS* and the insert containing the 1-kb downstream region of *lciE* were used for a four-way ligation, as described above, to generated plasmid pMLpUC18+0714Dw-Km-0716Dw (Data Set S1). This plasmid was digested with PvuII, and the resulting fragment was cloned into the pLAW344 allelic exchange vector digested with EcoRV to generate the plasmid pMLpLAW344-0714Dw-Km-0716Dw (Data Set S1).

To generate a clean deletion mutant in lpg2888, a 1-kb DNA fragment located on each side of the planned deletion was amplified by PCR using the primers listed in Data Set S1. The resulting plasmids were digested with the suitable enzymes, and the inserts were used for a three-way ligation into pUC-18 to generate pMW-18-Δlpg2888-3W (Data Set S1). This plasmid was digested with PvuII, and the resulting fragment was cloned into the pGY100 allelic exchange vector digested with XmnI to generate the plasmid pMW-100-Δlpg2888 (Data Set S1). The clean and marked allelic exchange deletion mutants were constructed as previously described (83, 85).

For the construction of the plasmid expressing the His-tagged LciR, the *lciR* gene was amplified by PCR using the primers listed in Data Set S1, cloned into pET-15b, and sequenced to generate the plasmid pML-pET15b+lpg0715 (Data Set S1).

### Bacterial growth in the presence of copper.

To determine the copper concentrations to be used in the β-galactosidase assays with both L. pneumophila and E. coli, the bacteria were grown in fresh AYE lacking Fe(NO_3_)_3_ or LB, respectively, with a wide range of copper concentrations, and the optical density at 600 nm (OD_600_) was determined in intervals of 1 h until reaching stationary phase. The same analysis was also performed with the other metals examined.

### β-Galactosidase assay.

β-Galactosidase assays were performed as previously described (19). L. pneumophila strains were grown for 48 h on charcoal-yeast extract (CYE) plates containing chloramphenicol (Cm). The bacteria were scraped off the plate and suspended in ACES-yeast extract (AYE) broth, and the bacterial OD_600_ was calibrated to 0.1 in fresh AYE lacking Fe(NO_3_)_3_, containing different concentrations of copper (or other metals, when indicated) and Cm. When other metals were used, 2 μM bathocuproine sulfonate (BCS) was added to the medium to adsorb any copper traces present in the stocks of the other metals. This concentration of BCS was also included when copper was used, and it did not affect the induction by copper. The resulting cultures were grown on a roller drum for about 18 h, until reaching an OD_600_ of about 3.2 (early stationary phase), and used for β-galactosidase assay.

β-Galactosidase assays in E. coli were performed similarly, but the E. coli strains were grown for about 6 h in LB containing different concentrations of copper, until reaching an OD_600_ of about 2.5 (early stationary phase), and used for β-galactosidase assay. The assays were done for 20, 50, or 100 μl of culture, and the substrate for β-galactosidase hydrolysis was *o*-nitrophenyl-β-d-galactopyranoside.

### Protein purification and gel mobility shift assay.

His_6_-LciR was purified from E. coli BL21(DE3) using nickel bead columns (Qiagen) according to the manufacturer’s instructions. After purification, the fractions containing the protein were dialyzed overnight against a buffer containing 10 mM Tris-HCl (pH 7.5), 5 mM MgCl_2_, 50 mM KCl, 0.1 mM EDTA, and 0.1 mM dithiothreitol. Glycerol was added to a concentration of 50%, and the purified protein was then stored at –20°C. A gel mobility shift assay was performed as previously described (32), with a few modifications. The regulatory region *lciE* (176 bp) was amplified by PCR using the primers listed in Data Set S1 and 3′ end labeled with digoxigenin (DIG) by using DIG-11-ddUTP (Roche). Increasing amounts of the purified His_6_-LciR protein (between 35 and 280 nM) were mixed with 0.75 nM the DIG-labeled probe in buffer containing 10 mM Tris-HCl (pH 7.5), 5 mM MgCl_2_, 50 mM KCl, 0.1 mM EDTA, 0.1 mM dithiothreitol, 250 μg/ml bovine serum albumin, and 50 μg/ml herring sperm DNA. For the competition experiments, a 100-fold excess of the unlabeled probe or mutated unlabeled probe was allowed to bind the His_6_-LciR protein for 30 min before addition of the DIG-labeled probe. The binding reaction was carried out for 30 min at room temperature, and samples then were loaded onto a 5% polyacrylamide–0.25× Tris-acetate-EDTA gel in 0.5× Tris-acetate-EDTA running buffer. Following electrophoresis, the gel was transferred to a nylon membrane and fixed by UV cross-linking. The DIG-labeled DNA fragments were detected by following the manufacturer’s instructions (Roche).

### Intracellular growth assays.

Intracellular growth assays of L. pneumophila strains in A. castellanii and HL-60-derived human macrophages were performed as previously described (86). Intracellular competition assays of L. pneumophila strains in A. castellanii also were performed as previously described (22).

### Reconstruction of phylogenetic trees.

Trees were reconstructed on the basis of concatenated alignments of the three proteins indicated for each tree. The trees were reconstructed using RAxML (87) under the LG + GAMMA evolutionary model with 100 bootstrap resampling.
